# Prognostic value of the association between MHC class I downregulation and PD-L1 upregulation in head and neck squamous cell carcinoma patients

**DOI:** 10.1038/s41598-019-44206-2

**Published:** 2019-05-22

**Authors:** Shin Hye Yoo, Bhumsuk Keam, Chan-Young Ock, Sehui Kim, Buhm Han, Ji-Won Kim, Keun-Wook Lee, Yoon Kyung Jeon, Kyeong Cheon Jung, Eun-Jae Chung, Seong Keun Kwon, Soon-Hyun Ahn, Myung-Whun Sung, Dae Seog Heo

**Affiliations:** 1 Department of Internal Medicine, Seoul National University Hospital, Seoul, Republic of Korea; 2 Cancer Research Institute, Seoul National University College of Medicine, Seoul, Republic of Korea; 3 Department of Pathology, Seoul National University Hospital, Seoul, Republic of Korea; 4 Department of Biomedical Sciences, Seoul National University College of Medicine, Seoul, Republic of Korea; 5 Department of Internal Medicine, Seoul National University Bundang Hospital, Seoul National University College of Medicine, Seongnam-si, Gyeonggi-do, Republic of Korea; 6 Department of Otorhinolaryngology, Seoul National University Hospital, Seoul, Republic of Korea

**Keywords:** Head and neck cancer, Tumour immunology

## Abstract

The purpose of this study was to evaluate the prognostic impact of major histocompatibility complex (MHC) class I expression and programmed death-ligand 1 (PD-L1) expression in patients with head and neck squamous cell carcinoma (HNSCC). A total of 158 patients with HNSCC were evaluated retrospectively. The expression of MHC class I and PD-L1 was analyzed in tumor specimens using immunohistochemistry. The association between MHC class I/PD-L1 expression and clinical outcome was evaluated by Kaplan-Meier and Cox regression analyses. Among 158 patients, 103 (65.2%) showed positive PD-L1 expression, and 20 (12.7%) showed no detectable expression of MHC class I. The frequency of PD-L1 positive expression with concomitant MHC class I loss was 7.0%. In the PD-L1-positive group, MHC class I loss was associated with a significantly worse survival compared with MHC class I positivity (median overall survival 39.3 months vs. not reached; *P* = 0.005), whereas MHC class I status provided no prognostic impact in the PD-L1 negative group. Neither PD-L1 nor MHC class I alone showed a significant difference in overall survival. The loss of MHC class I expression in PD-L1-positive HNSCC was associated with a poor clinical outcome. This suggested that MHC class I expression status might be useful for the prognosis of tumor progression in HNSCC when combined with PD-L1 expression status. External validation with enough numbers of participants in such subgroup should be needed for validation.

## Introduction

Head and neck squamous cell carcinoma (HNSCC) is the sixth most common malignancy in the world^1^ and is a heterogeneous disease entity arising from various anatomic sites, including the oral cavity, oropharynx, hypopharynx, and larynx. Recently, programmed death-1 (PD-1)/programmed death-ligand 1 (PD-L1) blockade has produced remarkably durable clinical responses in HNSCC, with an objective response rate (ORR) of 13.3%, median progression-free survival (PFS) of 2.0 months, and median overall survival (OS) of 7.5 months^2^.

PD-1 is a transmembrane immune inhibitory receptor that plays the role of self-tolerance in immune response by repressing the effector functions of T cells within the tumor microenvironment. The PD-1 ligand, PD-L1, delivers its inhibitory signals to PD-1-positive T-cells to suppress their cytotoxic activity by cell-intrinsic inhibition of antigen-specific signaling^3,4^. PD-1/PD-L1 interaction normally regulates the activation and termination of immune response, but some tumors express PD-L1 and use this interaction as one of the major immune escape mechanisms. Thus, this interaction has become an immunologic therapeutic target in various malignancies^5^. Furthermore, PD-L1 expression was found to be positively correlated with ORR and PFS, suggesting that PD-L1 expression might be a positive predictive marker in some tumors^6,7^. Despite these encouraging results, a significant portion of PD-L1-positive patients with advanced HNSCC do not respond to these immune checkpoint inhibitors (ICIs)^8^. This suggests that there exist additional escape mechanisms that allow tumor cells blocked by ICIs to avoid attack by cytotoxic T lymphocytes.

It is well established that tumor cells often harbor a loss or down-regulation of the major histocompatibility complex (MHC) class I molecules on their surface, and this is considered an immune escape mechanism of the tumor^9^. The main function of MHC class I molecules is to display peptides, including tumor-associated antigens, to cytotoxic CD8 + T cells. Defects in MHC class I molecules lead to impaired T-cell mediated cytotoxicity against tumor cells. The deregulated expression of MHC class I genes has been demonstrated in various tumor types^10–13^. Particularly, the total loss of MHC class I expression in primary and metastatic HNSCC lesions occurred in approximately 15% and 40%, respectively^14^. However, although some studies have revealed that MHC class I down-regulation might be a poor prognostic factor in HNSCC^15–17^, the prognostic relevance of MHC class I loss remains unclear.

Although MHC class I loss is a frequent event and is thought to confer a tumor escape function, little is known about its clinical significance in PD-L1-positive patients with HNSCC. In the present study, we first analyzed the associative expression patterns of MHC I and PD-L1 in patients with locally advanced HNSCC. We then sought to explore the prognostic significance of impaired antigen presentation caused by MHC I loss combined with positive PD-L1 expression.

## Results

### Baseline demographic and clinical characteristics of HNSCC patients

The baseline characteristics of the patients and their tumors are described in Table 1. Male patients were 72.1%, and median age was 59 years (range, 20 to 89 years). A majority of the patients (96.2%) had the Eastern Cooperative Oncology Group performance status of 0 or 1. Ninety-six patients (60.8%) were classified as never smokers. Most of primary tumors were located in the oropharynx (41.8%) and oral cavity (32.9%). All patients were evaluated for p16 expression, which was strongly positive in 56 patients (35.4%).

**Table 1:** Baseline demographic and clinical characteristics of 158 patients with locally advanced head and neck cancer.

Variables	Detail	*n*	%
**Patient factors**			
Age at diagnosis	Median (range)	59 (20–89)	
	≥60	57 (43.0)	
	<60	90 (57.0)	
Sex	Male	114	72.1
	Female	44	27.9
ECOG PS at diagnosis	0	86	54.4
	1	66	41.8
	≥2	6	3.8
Smoking	Current or ex-smoker	56	36.8
	Mean PY ± SD	29.8 ± 17.2	
	Never smoker	96	63.2
**Tumor factors**			
Location of tumor	Oropharynx	66	41.8
	Hypopharynx	12	7.6
	Larynx	14	8.9
	Nasopharynx	1	0.6
	Oral cavity	52	32.9
	Nasal cavity	4	2.5
	Others	9	5.7
T classification	0	4	2.5
	1	35	22.4
	2	60	38.5
	3	21	13.5
	4	36	23.1
N classification	0	46	29.3
	1	35	22.3
	2	72	45.9
	3	4	2.5
AJCC 7th stage	I	13	8.3
	II	18	11.4
	III	32	20.4
	IV	94	59.9
**Treatment factors**			
Induction chemotherapy	Yes	36	22.8
	No	122	77.2
Type of definite Tx	CCRT (or RT)	36	22.8
	Surgery	122	77.2
Adjuvant Tx	Yes	89	56.3
	RT	51	32.3
	CCRT	38	24.0
	No	69	43.7
**Pathology factors**			
Differentiation	P/D	34	21.5
	M/D	59	37.3
	W/D	44	27.9
	Others	21	13.3
p16	Negative/Weak positive	102	64.6
	Strong Positive	56	35.4

*ECOG* the Eastern Cooperative Oncology Group, *PS* performance status, *PY* pack-year, *SD* standard deviation, *AJCC* American Joint Committee on Cancer, *CCRT* concurrent chemoradiation treatment, *RT* radiation treatment, *Tx* treatment, *P/D* poorly differentiated, *M/D* moderately differentiated, *W/D* well-differentiated, *HPV* human papillomavirus, *PCR* polymerase chain reaction;

Missing data was as follows: 6 for smoking, 2 for T classification, 1 for N classification, and 1 for AJCC stage.

### Classification of HNSCC patients according to MHC class I and PD-L1 expression

**Figure 1:**
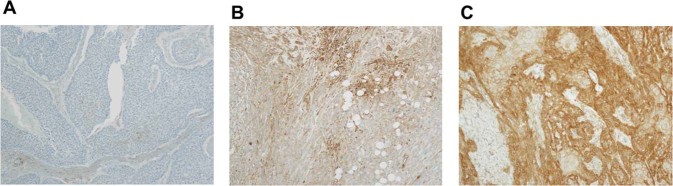
Major histocompatibility complex (MHC) class I and programmed death-ligand 1 expression in head and neck squamous cell carcinoma patients. Representative immunohistochemical staining for MHC class I on paraffin. MHC class I expression is undetectable (**A**), weak (**B**), or strong (**C**).

All patients were analyzed for MHC class I and PD-L1 expression, as shown in Table 2. The immunohistochemical analysis showed 20 tumors (12.7%) with no detectable expression of MHC class I molecule, 50 tumors (31.6%) with weak expression, and 88 tumors (55.7%) with strong expression. Representative immuno-staining patterns of MHC class I expression are shown in Fig. 1A–C. PD-L1 expression patterns were also examined in this cohort. PD-L1 expression was positive in a majority of patients (65.2%) and negative in 55 patients (34.8%). Among PD-L1-positive patients, MHC class I expression was absent in 11 (10.7%) and weak in 29 (28.1%) patients.

**Table 2:** The number of patients according to the correlated expressions for MHC class I and PD-L1.

		PD-L1		
		Negative	Positive	Total
MHC class I	None	9	11	20 (12.7)
	Weak	21	29	50 (31.6)
	Strong	25	63	88 (55.7)
	Total	55 (34.8)	103 (65.2)	158

*HNSCC* head and neck squamous cell carcinoma, *PD-L1* programmed death-ligand 1, *MHC* major histocompatibility complex.

The associations of MHC class I and PD-L1 expression with other clinico-pathological factors were also analyzed. No significant association was detected between PD-L1 or MHC class I expression and other clinico-pathological factors (Supplementary Tables S1 and S2).

### Survival outcomes in relation to PD-L1 and MHC class I status

**Figure 2:**
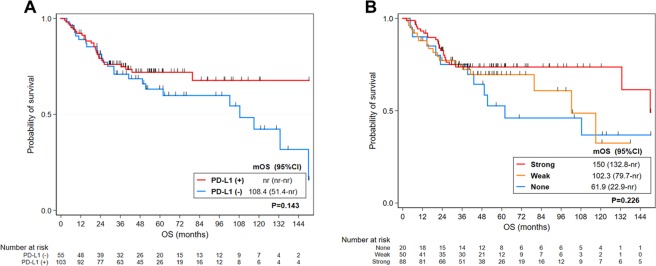
Kaplan-Meier curves for overall survival (OS) in patients with head and neck squamous cell carcinoma (HNSCC). OS stratified by programmed death-ligand 1 expression status (**A**) and OS stratified by major histocompatibility complex class I expression status (**B**). *mOS* median overall survival, *nr* not reached.

To evaluate the prognostic value of PD-L1 and MHC class I expression in patients with HNSCC, the survival outcomes were analyzed according to the PD-L1 and MHC class I status. The median follow-up time was 54.5 months (range, 2.2 to 327.6 months). The Kaplan-Meier estimates of survival for PD-L1 negative and positive patients are shown in Fig. 2A. According to the Kaplan-Meier survival curves, PD-L1-negative patients trended toward a poor survival, although this was not significant (*P* = 0.143). Likewise, MHC class I status did not make a significant difference in OS in HNSCC (Fig. 2B). This suggests that neither PD-L1 nor MHC class I alone had a prognostic significance in HNSCC.

**Figure 3:**
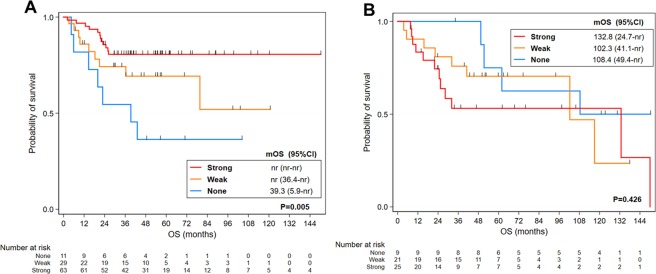
Kaplan-Meier curves for overall survival (OS) according to PD-L1 status in head and neck squamous cell carcinoma (HNSCC) patients stratified by MHC class I expression. OS stratified by major histocompatibility complex (MHC) class I expression in programmed death-ligand 1 (PD-L1)-positive HNSCC patients (**A**) and OS stratified by MHC class I expression in PD-L1-negative HNSCC patients (**B**). *mOS* median overall survival, *nr* not reached.

When analyzed by MHC class I expression in the PD-L1-positive group, patients with MHC class I loss showed a significantly worse survival (*P* = 0.005), with a median OS of 39.3 months compared with MHC class I-positive patients (Fig. 3A). In contrast, MHC class I status had no prognostic impact in PD-L1-negative patients (Fig. 3B).

### Factors associated with overall survival

According to a Cox univariate proportional hazards analysis, negative MHC class I expression was significantly associated with reduced OS in the PD-L1-positive group (HR = 4.24, 95% CI 1.66–10.78; *P* = 0.002). Loss of p16 expression, advanced T classification (T3-T4 versus T1-T2), and receipt of (chemo)radiotherapy as definitive treatment were also associated with poor OS (Table 3). In multivariate analysis, MHC class I loss in the PD-L1-positive setting retain prognostic significance (HR = 3.84, 95% CI 1.43–10.35; *P* = 0.008), as well as loss of p16 expression was associated with a worse survival (HR = 3.12, 95% CI 1.12–8.68; *P* = 0.029).

**Table 3:** Univariate and multivariate Cox regression analysis of OS among HNSCC patients.

		OS in PD-L1 (+) subgroup						OS in PD-L1 (−) subgroup					
		Univariate			Multivariable			Univariate			Multivariable		
Variables	Detail	HR	95% CI	*P* value	aHR	95% CI	*P* value	HR	95% CI	*P* value	aHR	95% CI	*P* value
**Pathology factors**													
MHC class I	Weak (ref: Strong)	1.99	0.84–4.72	0.120	2.34	0.82–6.63	0.111	0.68	0.27–1.71	0.415	0.78	0.30–2.00	0.600
	None (ref: Strong)	4.24	1.66–10.78	0.002	3.84	1.43–10.35	0.008	0.48	0.15–1.56	0.225	0.47	0.13–1.74	0.261
Differentiation	P/D (ref: non-P/D)	0.41	0.12–1.35	0.141	*not entered*			1.81	0.70–4.65	0.219	*not entered*		
p16	(−) (ref: (+))	2.37	1.00–5.58	0.049	3.12	1.12–8.68	0.029	1.85	0.63–5.48	0.265	*not entered*		
**Patient factors**													
Age at Dx	≥60 (ref: <60)	2.09	0.99–4.42	0.055	1.14	0.46–2.81	0.775	1.44	0.61–3.44	0.406	*not entered*		
Sex	Female (ref: Male)	0.84	0.37–1.91	0.683	*not entered*			1.25	0.46–3.40	0.664	*not entered*		
ECOG PS at Dx	1–2 (ref: 0)	2.05	0.97–4.33	0.061	1.08	0.44–2.68	0.863	2.64	1.04–6.71	0.041	2.23	0.82–6.07	0.118
Smoking	Smoker (ref: Never)	0.96	0.43–2.18	0.930	*not entered*			1.80	0.76–4.27	0.183	*not entered*		
**Tumor factors**													
Location of tumor	Non-OPx (ref: OPx)	1.83	0.84–3.97	0.127	*not entered*			2.66	0.79–9.02	0.116	*not entered*		
T classification	T3-T4 (ref: T0-T2)	2.26	1.07–4.75	0.032	0.72	0.23–2.26	0.568	2.14	0.92–4.97	0.077	1.80	0.71–4.52	0.214
N classification	N2-N3 (ref: N0-N1)	1.46	0.69–3.08	0.324	*not entered*			1.83	0.80–4.18	0.153	*not entered*		
**Treatment factors**													
Induction Ctx	Yes (ref: No)	2.04	0.94–4.43	0.072	1.18	0.43–3.20	0.751	0.61	0.20–1.91	0.396	*not entered*		
Type of definite Tx	RTx (ref: Surgery)	2.74	1.29–5.82	0.009	2.71	0.95–7.71	0.061	1.55	0.61–3.95	0.359	*not entered*		

*OS* overall survival, *HNSCC* head and neck squamous cell carcinoma, *PD-L1* programmed death-ligand 1, *HR* hazard ratio, *aHR* adjusted hazard ratio, *CI* confidence interval, *ref* reference, *MHC* major histocompatibility complex, *P/D* poorly differentiated, *Dx* diagnosis, *ECOG* the Eastern Cooperative Oncology Group, *PS* performance status, *OPx* oropharynx, *CTx* chemotherapy, *Tx* treatment, *CCRT* concurrent chemoradiation treatment, *RT* radiation treatment

Meanwhile, MHC class I loss in the PD-L1-negative setting did not retain any prognostic significance. Poor performance status was significantly associated with poor survival in the PD-L1-negative setting in univariate analysis (HR = 2.64, 95% CI 1.04–6.71; *P* = 0.041), but it trended toward a worse survival without prognostic significance (HR = 2.23, 95% CI 0.82–6.07; *P* = 0.118).

## Discussion

Although MHC class I loss has been reported in several types of tumors ranging from 15% to 96%^12,13,18,19^, few studies have assessed the proportion of concomitant expression of MHC class I and PD-L1, whereas some have analyzed a single parameter’s expression and relation with immune cell infiltration only^20–22^. Our data suggested that the frequency of MHC class I loss with concurrent PD-L1 expression was approximately 7.0% in our tissue microarray cohort, which is similar to 7% in non-small cell lung cancer^23^ and 4.3% in conjunctival melanoma^24^. However, the prevalence is higher in esophageal squamous cell carcinoma (12.2%)^25^, non-small cell lung cancer (17%)^26^, and hepatocellular carcinoma (17%)^27^. This discrepancy might be due to a cutoff point of the high/low expression of MHC class I, or differences in tumor types. For example, in the former case^25,27^, MHC class I expression is classified as low expression and high expression groups by using Allred scoring, not as absent or present. On the other hand, various results might result from the small number of participants in studies examining the proportion of MHC class I loss and PD-L1 expression including ours. Nevertheless, we found that some HNSCC patients lose MHC class I expression even with a high level of PD-L1 expression; this pattern is also seen in patients with other tumor types^23–27^.

The down-regulation of MHC class I alone has been controversial as a prognostic marker. For example, some studies demonstrated that MHC class I down-regulation was associated with improved survival in lung cancer and breast cancer, whereas others reported the opposite outcome in the same types of cancers^28–30^. This discrepancy of the results might be explained by our findings in HNSCC that the loss of MHC class I on tumors is a prognostic marker of poor survival only when PD-L1 is concomitantly expressed. According to Perea *et al*.^26^, non-small cell lung cancer patients with decreased MHC class I and high PD-L1 expression have larger tumor sizes that show a more aggressive phenotype, similar to esophageal cancer patients^25^. This association is not observed in patients with only MHC class I downregulation or PD-L1 upregulation. Although there was a lack of information on survival outcomes in Perea *et al*. that could limit the interpretation, the obtained results could explain the poor survival in patients with MHC class I loss in PD-L1-positive tumors as shown in our study. In addition, the most striking finding from the study by Perea *et al*.^26^ is that the proportion of CD8-positive T cell infiltration was 100% in PD-L1-positive patients with high MHC class I expression compared to 37% in PD-L1-positive patients with MHC class I loss, which indicates that T-cell-mediated adaptive immune response could not be activated without the presence of MHC class I expression. MHC class I might play a crucial role in an immune escape mechanism of the adaptive immune response exerted by CD8 + T cells, which induces the up-regulation of PD-L1 through IFN-gamma^3^. Based on this assumption, we cautiously postulate that patients with MHC class I loss would have low expression of T cell infiltration, induce little adaptive immune response, and show poor clinical outcomes in PD-L1 positive setting despite lack of information on CD8-positive T cell infiltration in our study.

However, Perea and colleagues present some differences in T cell infiltration by MHC class I expression in PD-L1-negative patients, although the magnitude of differences is smaller than those in PD-L1-positive patients. We suggest that a different mechanism than T cell infiltration might compensate for the differences in intra-tumoral T cells and promote tumor progression in PD-L1-negative tumors^31^. Our result is slightly different from a previous study of classical Hodgkin’s lymphoma in which patients with decreased/absent MHC I expression had worse survival regardless of PD-L1 positivity^30^. This inconsistent result emphasizes the heterogeneity of cancer. PD-L1 expression was also constitutive in the previous study of Hodgkin lymphoma rather than regulated by an adaptive immune response^32^. Furthermore, our findings are not in line with results from studies on esophageal cancer and hepatocellular carcinoma^25,27^. According to Ito *et al*.^25^, esophageal cancer patients with both high expression of PD-L1 and MHC class I seem to have a worse prognosis than patients with high expression of PD-L1 and decreased MHC class I expression. On the other hand, in patients with hepatocellular carcinoma^27^, MHC class I downregulation appears to be associated with poor prognosis among the low PD-L1 group, whereas no significant difference in survival is observed according to MHC class I expression among the high PD-L1 group. These two studies do not focus on the prognostic role of MHC class I loss in regard to PD-L1 upregulation, but focus on PD-L1’s role in the presence of MHC class I expression. Despite the lack of explanation regarding these findings due to differences among the study aims, we assume that the impact of MHC class I expression in correlation with PD-L1 expression on survival might be different between cancer types. Every tumor type has a different escape mechanism, and therefore it is unique that we found differences in prognosis by MHC class I loss and concomitant PD-L1 expression in HNSCC patients. Yet, the speculation related to the association of CD8-positive T cell infiltration with MHC class I expression remains to be validated.

PD-L1 expression has been reported in several malignancies including HNSCC, implying its vital role in the process of tumor escape through PD-1 and PD-L1 interaction. Two study groups reported that PD-L1 positivity is a poor prognostic factor with a shortened OS in HNSCC^33,34^. Another group showed that PD-L1 expressed on the immune cell surface is associated with a good prognosis^35^. In addition, a recent study suggested that PD-L1 on tumor cells is closely associated with a longer disease-free survival^36^. The results were inconsistent and conflicting. This inconsistency reflects that the host immune surveillance is acting through multiple pathways^4^. For example, in addition to cell-intrinsic inhibition of antigen-specific signaling mediated by PD-1/PD-L1 interaction, CTLA-4 suppresses CD28-mediated T cell activation by competitive binding with CD80 and CD86^37^. Furthermore, there are several immune inhibitory molecules such as CTLA-4, LAG-3, TIM-3, and TIGIT with unique functions and different tissue sites^38^. Besides, metabolic reprogramming by tumor microenvironment^39^ and regulation of other anabolic and catabolic pathway by mitochondria^40^ also influences tumor-immune response as well as epigenetic modification of T cells^41^. Therefore, the prognostic impact of PD-L1 might be complicated by these various factors.

MHC class I might be important in immune surveillance only in tumors in which the adaptive immune response plays a role. In this context, immune checkpoint inhibitors might not be effective in PD-L1-positive tumors harboring MHC class I loss. Further study on a larger population size is needed to draw conclusions on the clear role of MHC class I in the prognosis of PD-L1-positive HNSCC patients. On the other hand, since treatment strategy may vary depending on the concept of ‘hard loss’ and ‘soft loss’^12^, discriminating the type of MHC class I by mechanisms is important. However, there is a discrepancy between losses of MHC class I expression detected by immunohistochemistry (IHC) and commonly characterized genetic alterations of MHC class I gene or antigen presenting machinery pathway such loss of heterozygosity (LOH) or mutations. LOH in chromosome 6 (human leukocyte antigen) or 15 (beta-2-microglobulin) is the most commonly found mechanism of MHC class I alteration^13^. However, some cases with loss of MHC class I expression present with other complex mechanisms and different phenotypes can occur^26,42^. As selective loss or allelic loss of MHC class I molecule might not induce the loss of expression and not be detected by IHC, they could be the reason for underestimating MHC class I loss. To minimize underestimation, more comprehensive diagnostic methods are needed including not only IHC, but also microsatellite analysis to detect LOH and sequencing to analyze copy number alterations and mutations. Furthermore, it is also important to distinguish genetic aberrations of MHC class I and its polymorphisms.

Our study has several limitations. First, this was a single-institutional retrospective study with a small population. The small size of subgroup with MHC class I loss and PD-L1 expression may limit the interpretation of our findings. Because further study with larger sample size can show different results, external validation with enough numbers of participants in such subgroup must be performed to validate our conclusion. Moreover, there could be a selection bias since the tissue microarray was made only from patients with sufficient tissue samples. Second, there are some issues about immunohistochemical detection of PD-L1. We used E1L3N, the rabbit monoclonal antibody for PD-L1 expression, which has been regarded as having relatively lower concordance rate than other antibodies for PD-L1 such as SP142 or SP263^43,44^. The aforementioned drawback associated with this antibody may be one of the limitations in our study. However, although various companion diagnostics for PD-L1 expression have been recently commercially available, in the early days those tests were not set up and E1L3N was widely used for research purposes. Furthermore, E1L3N showed good agreement rate with other antibodies^45^ in some studies performed in head and neck cancer patients^42–44^. Besides, we evaluated PD-L1 expression only on tumor cells, but not on immune cells. There is a report that the prognostic implication of surface marker expression might differ depending on the cell type on which the marker is expressed^35^. Thus, PD-L1 expressed on immune cells might affect the survival outcome in a different way from PD-L1 expressed on tumor cells. Third, we did not perform IHC of CD8-positive T cells, which is a key component for activated adaptive immune response. Although our speculation was based on evidence that CD8-positive T cell infiltration is positively correlated with MHC class I expression in several cancer types^22,26^ including HNSCC^45^, it is difficult to judge whether the adaptive immune response is indeed activated in PD-L1-positive tumors. Further studies are strongly needed to verify this association and survival differences. Finally, because our study did not evaluate the presence of LOH but examined MHC class I loss in terms of cell surface protein expression, we were unable to discriminate ‘hard loss’ and ‘soft loss’. Regarding this limitation, tests for identifying genetic alterations such as microsatellite markers has to be investigated in further analyses. Future studies to demonstrate the mechanism of MHC class I loss in HNSCC patients are also needed. Regardless of this limitation, our study has its own valuable point as a hypothesis generating study rather than confirmatory study, which shows the different prognostic role of MHC class I loss according to PD-L1 expression.

The current study demonstrates that loss of MHC class I expression is associated with a poor prognosis in PD-L1-positive HNSCC. This suggests that the combination of MHC class I and PD-L1 might be useful to better predict the clinical course of the disease. However, external validation should be needed to verify our conclusion. Moreover, these biomarkers require further validation, especially in HNSCC patients treated with ICIs.

## Methods

### Patients and data collection

We retrospectively reviewed the medical records of patients diagnosed with locally advanced HNSCC and treated at Seoul National University Hospital (SNUH) between December 2004 and December 2014. The eligibility criteria were as follows: HNSCC tumors were histologically confirmed by a pathologist according to the seventh edition of the American Joint Committee on Cancer; patients had to be 19 years or older; patients were treated initially with induction chemotherapy and/or concurrent chemoradiotherapy or radical surgery; sufficient formalin-fixed, paraffin-embedded tumor samples IHC were obtained. A total of 158 patients were enrolled. Baseline patient characteristics (including age, sex, the Eastern Cooperative Oncology Group performance status, smoking status, histologic differentiation, location of tumor, and stage), and treatment factors (including types of definite and adjuvant treatments) were retrospectively obtained from medical records. The study was approved by the Institutional Review Boards of SNUH (approval number: H-1710–075–893) and was conducted in accordance with the 1964 Helsinki declaration and its later amendments or comparable ethical standards. Informed consent was obtained from all individual participants included in the study.

### Immunohistochemistry

We used TMA of the patients included in the study for IHC staining. From representative tumor regions identified by H&E stained sections, tissue cylinders were obtained and arrayed into a TMA block. Immunohistochemical staining was conducted using serial sections from this TMA block using the following antibodies. In cases with information of PD-L1 expression by IHC, rabbit anti-PD-L1 (E1L3N) XP® mAb (Cell Signaling Technology, Danvers, MA, USA) was utilized with the Ventana Benchmark XT system (Ventana Medical Systems) at the Department of Pathology at SNUH. PD-L1 IHC based on the intensity and proportion of membrane staining in tumor cells was scored as follows: 0, less than 5% of tumor cells; 1, weak in ≥5% of tumor cells; 2, moderate in ≥5% of tumor cells; and 3, strong in ≥5% of tumor cells^46^. Those with a membranous staining score for PD-L1 more than 1 were regarded as positive for PD-L1. MHC class I expression was assessed by IHC with anti-HLA class I A, B, and C antibody (EMR8–5, Cosmo Bio, Tokyo, Japan). Based on the percentage of MHC class I-positive tumor cells, each patient was classified according to the criteria established by the Human Leukocyte Antigen and Cancer component of the 12^th^ International Histocompatibility Workshop^47^. When the cell membrane was stained as strong as stromal lymphocytes or endothelial cells in more than 75% of the tumor cells, expression levels were defined as strong (2); If heterogeneous membrane staining was found in more than 25% of the tumor cells, it was defined as weak (1); If less than 75% of the tumor cells lacked membrane staining, it was defined as none (0). p16 was evaluated using mouse anti-p16 (E6H4) monoclonal antibody (mAb) (Roche/MTM/Ventana Laboratories, Tucson, AZ, USA) and was considered positive in case of diffuse and strong nuclear and cytoplasmic staining in 70% or more of tumor cells^48^. All the IHC results were assessed by expert pathologists (KCJ, KSH) at SNUH who were blinded to the clinical and pathological data.

### Statistical analysis

The descriptive data of the patients are presented as numbers with percentages or mean values with standard deviations. The association of PD-L1 positivity and MHC class I loss with demographic and clinico-pathological variables was analyzed using Student’s t-test or chi-square tests, where appropriate. The outcome of interest was OS, which was defined as the time from the date of diagnosis to the date of death or the date of the last follow-up if censored. Survival outcome was analyzed using Kaplan-Meier estimation. We performed the log-rank test to compare OS according to PD-L1 and MHC class I. To identify the impact of MHC class I on OS by PD-L1 expression, we performed subgroup analyses by stratification. In addition, factors associated with OS were analyzed using univariate Cox regression analyses separately for PD-L1-positive and PD-L1-negative patients. We performed multivariable Cox regression analysis employing statistically significant variables from the univariate analysis (*P* < 0.10). Although MHC class I loss was insignificant in univariate analysis, we included it in the multivariable analysis because we regarded it as a clinically essential variable. Statistical significance was defined as *P* < 0.05. All statistical tests were two-sided and were performed using STATA, version 15 software (StataCorp LP, College Station, TX, USA).

## Supplementary information

Supplementary Table S1, Supplementary Table S2

## Data Availability

The datasets generated during and/or analysed during the current study are available from the corresponding author on reasonable request.
