# Supplementary material for: Prognostic value of the association between MHC class I downregulation and PD-L1 upregulation in head and neck squamous cell carcinoma patients
Source: Sci Rep. 2019 May 22;9:7680. doi: 10.1038/s41598-019-44206-2 (PMC6531443; doi:10.1038/s41598-019-44206-2)
Supplement: Supplementary file 1 — Supplementary Table S1, Supplementary Table S2 [file 41598_2019_44206_MOESM1_ESM.docx]

**Prognostic value of the association between MHC class I downregulation and PD-L1 upregulation in head and neck squamous cell carcinoma patients**

Shin Hye Yoo^1^, Bhumsuk Keam^1,2*^, Chan-Young Ock^1^, Sehui Kim^3^, Buhm Han^4^, Ji-Won Kim^5^, Keun-Wook Lee^5^, Yoon Kyung Jeon^3^, Kyeong Cheon Jung^3^, Eun-Jae Chung^6^, Seong Keun Kwon^6^, Soon-Hyun Ahn^6^, Myung-Whun Sung^6^, and Dae Seog Heo^1,2^

^1^Department of Internal Medicine, Seoul National University Hospital, Seoul, Republic of Korea;

^2^Cancer Research Institute, Seoul National University College of Medicine, Seoul, Republic of Korea;

^3^Department of Pathology, Seoul National University Hospital, Seoul, Republic of Korea;

^4^Department of Biomedical Sciences, Seoul National University College of Medicine, Seoul, South Korea

^5^Department of Internal Medicine, Seoul National University Bundang Hospital, Seoul National University College of Medicine, Seongnam-si, Gyeonggi-do, Republic of Korea;

^6^Department of Otorhinolaryngology, Seoul National University Hospital, Seoul, Republic of Korea;

**Supplementary Table S1. Association of MHC class I loss with baseline characteristics**

| Variables | Detail | MHC class I  None | MHC class I  Weak/Strong | *P* value |
| --- | --- | --- | --- | --- |
| **Patient factors** |  |  |  |  |
| Age at diagnosis | <60 | 11 (12.2) | 79 (87.8) | 0.850 |
|  | ≥60 | 9 (13.2) | 59 (86.8) |  |
| Sex | Male | 18 (15.8) | 96 (84.2) | 0.065 |
|  | Female | 2 (4.6) | 42 (95.4) |  |
| ECOG PS at diagnosis | 0 | 7 (8.1) | 79 (91.9) | 0.062 |
|  | ≥1 | 13 (18.1) | 59 (81.9) |  |
| Smoking | Current or ex | 5 (8.9) | 51 (91.1) | 0.239 |
|  | Never | 15 (15.6) | 81 (84.4) |  |
| **Tumor factors** |  |  |  |  |
| Location of tumor | Oropharynx | 5 (7.6) | 61 (92.4) | 0.104 |
|  | Non-Oropharynx | 15 (16.3) | 77 (83.7) |  |
| T classification | 0-2 | 8 (8.1) | 91 (91.9) | 0.075 |
|  | 3-4 | 10 (17.5) | 47 (82.5) |  |
| N classification | 0-1 | 6 (7.4) | 75 (92.6) | 0.063 |
|  | 2-3 | 13 (17.1) | 63 (82.9) |  |
| AJCC 7th stage | I-III | 4 (6.4) | 59 (93.6) | 0.070 |
|  | IV | 15 (16.0) | 79 (84.0) |  |
| **Pathology factors** |  |  |  |  |
| Differentiation | P/D | 4 (11.8) | 30 (88.2) | 0.860 |
|  | non-P/D | 16 (12.9) | 108 (87.1) |  |
| p16 | Negative/weak positive | 12 (11.8) | 90 (88.2) | 0.648 |
|  | Strong positive | 8 (14.3) | 48 (85.7) |  |

*MHC* major histocompatibility complex, *ECOG* Eastern Cooperative Oncology Group, *PS* performance status, *AJCC* American Joint Committee on Cancer, *P/D* poorly differentiated

Missing data for the table was as follows: 6 for smoking status, 2 for T classification, 1 for N classification, and 1 for AJCC stage.

**Supplementary Table S2. Association of PD-L1 positivity with baseline characteristics**

| Variables | Detail | PD-L1 (-) | PD-L1 (+) | *P* value |
| --- | --- | --- | --- | --- |
| **Patient factors** |  |  |  |  |
| Age at diagnosis | <60 | 32 (35.6) | 58 (64.4) | 0.821 |
|  | ≥60 | 23 (33.8) | 45 (66.2) |  |
| Sex | Male | 44 (38.6) | 70 (61.4) | 0.108 |
|  | Female | 11 (25.0) | 33 (75.0) |  |
| ECOG PS at diagnosis | 0 | 26 (30.2) | 60 (69.8) | 0.187 |
|  | ≥1 | 29 (40.3) | 43 (59.7) |  |
| Smoking | Current or ex-smoker | 19 (33.9) | 37 (66.1) | 0.753 |
|  | Never | 35 (36.5) | 61 (63.5) |  |
| **Tumor factors** |  |  |  |  |
| Location of tumor | Oropharynx | 18 (27.3) | 48 (72.7) | 0.092 |
|  | Non-Oropharynx | 37 (40.2) | 55 (59.8) |  |
| T classification | 0-2 | 32 (32.3) | 67 (67.7) | 0.566 |
|  | 3-4 | 21 (36.8) | 36 (63.2) |  |
| N classification | 0-1 | 31 (38.3) | 50 (61.7) | 0.291 |
|  | 2-3 | 23 (30.3) | 53 (69.7) |  |
| AJCC 7th stage | I-III | 26 (41.3) | 37 (58.7) | 0.138 |
|  | IV | 28 (29.8) | 66 (70.2) |  |
| **Pathology factors** |  |  |  |  |
| Differentiation | P/D | 12 (35.3) | 22 (64.7) | 0.947 |
|  | non-P/D | 43 (34.7) | 81 (65.3) |  |
| p16 | Negative/weak positive | 39 (38.2) | 63 (61.8) | 0.223 |
|  | Strong positive | 16 (28.6) | 40 (71.4) |  |

*PD-L1* programmed death-ligand 1, *ECOG* Eastern Cooperative Oncology Group, *PS* performance status, *AJCC* American Joint Committee on Cancer, *P/D* poorly differentiated

Missing data for the table was as follows: 6 for smoking status, 2 for T classification, 1 for N classification, and 1 for AJCC stage.
